# Neural correlates of multi-day learning and savings in sensorimotor adaptation

**DOI:** 10.1038/s41598-018-32689-4

**Published:** 2018-09-24

**Authors:** M. F. L. Ruitenberg, V. Koppelmans, Y. E. De Dios, N. E. Gadd, S. J. Wood, P. A. Reuter-Lorenz, I. Kofman, J. J. Bloomberg, A. P. Mulavara, R. D. Seidler

**Affiliations:** 1 School of Kinesiology, University of Michigan, Ann Arbor, MI USA; 2 KBRwyle Science, Technology, and Engineering Group, Houston, TX USA; 3 NASA Johnson Space Center, Houston, TX USA; 4 Department of Psychology, University of Michigan, Ann Arbor, MI USA; 5 Present Address: Department of Experimental Psychology, Ghent University, Ghent, Belgium; 6 Present Address: Department of Psychiatry, University of Utah, Salt Lake City, UT USA; 7 Present Address: Department of Applied Physiology & Kinesiology, University of Florida, Gainesville, FL USA

## Abstract

In the present study we evaluated changes in neural activation that occur over the time course of multiple days of sensorimotor adaptation, and identified individual neural predictors of adaptation and savings magnitude. We collected functional MRI data while participants performed a manual adaptation task during four separate test sessions over a three-month period. This allowed us to examine changes in activation and associations with adaptation and savings at subsequent sessions. Participants exhibited reliable savings of adaptation across the four sessions. Brain activity associated with early adaptation increased across the sessions in a variety of frontal, parietal, cingulate, and temporal cortical areas, as well as various subcortical areas. We found that savings was positively associated with activation in several striatal, parietal, and cingulate cortical areas including the putamen, precuneus, angular gyrus, dorsal anterior cingulate cortex (dACC), and cingulate motor area. These findings suggest that participants may learn how to better engage cognitive processes across days, potentially reflecting improvements in action selection. We propose that such improvements may rely on action-value assignments, which previously have been linked to the dACC and striatum. As correct movements are assigned a higher value than incorrect movements, the former are more likely to be performed again.

## Introduction

Sensorimotor adaptation refers to the ability to adjust our behavior to changing environmental or internal demands to maintain appropriate, goal-directed motor performance. During the early phase of such adaptation, which is thought to rely on cognitive processes, fast improvements are often observed. The later phase of adaptation, in which automaticity develops, is characterized by slower improvements^1–3^. Individual differences in the rate of adaptation have been related to variability in visuospatial working memory abilities. For example, several studies found that better visuospatial working memory was associated with faster rates of manual adaptation early in practice^1,4,5^. This corroborates that the early, but not late, phase of adaptation involves visuospatial cognitive processes. Another study examined the mechanism underlying the contribution of working memory to adaptability^6^. The results demonstrated that the beneficial effect of a high visuospatial working memory capacity on adaptation was related to a subject’s ability to use an explicit strategy during adaptation.

Studies have shown that participants adapt faster when they have previously been exposed to the same perturbation. This indicates that changes in motor representations after adaptation can outlast the training session. Indeed, savings of adaptation have been observed one day after initial learning^7–9^, one month later^10^, five months later^11^, and even as much as one year after initial learning^12,13^. Behavioral studies suggest that in manual adaptation tasks, such savings may rely on the explicit recall of the successful strategy to deal with the perturbation during initial adaptation. For example, one study^14^ observed that savings one day after initial learning was restricted to explicit components of adaptation, which are thought to reflect cognitive processes such as action selection and working memory. In addition, another study^15^ observed that within-session savings occurred only when participants adapted to relatively large perturbations, which, in comparison to smaller perturbations, are more likely to be explicitly noticed (45° vs. 15° rotated feedback). Again, savings was observed for explicit aiming components but not implicit error-based measures of adaptation. Another way to examine savings is to measure astronauts’ performance after landing when they readjust to Earth’s gravity. For example, it has been found that faster improvements during the early phase of a locomotor adaptation task directly after flight were predictive of faster recovery (i.e., more savings) across several test sessions in an approximately one-month period post flight^16^. Taken together these studies indicate that savings after an initial practice session may reflect a declarative rather than procedural form of memory, relying on recall of a previously successful adaptation strategy, which facilitates action selection. This implies that the same mechanism may underlie early adaptation and multi-day savings. Thus, brain mechanisms that are known to play a role in early adaptation may also be involved in long-term savings. While recall of the adaptation strategy is thus thought to be explicit, the cognitive processes contributing to this strategy could be either implicit or explicit in nature.

So far, few studies have examined brain mechanisms associated with multi-day savings in sensorimotor adaptation. One study^12^ investigated how structural brain changes, as measured by magnetic resonance imaging (MRI) after one week of training (42 minutes of training per day), contribute to long-term memory of manual adaptation one year later. They observed that training led to increased gray matter concentration in participants’ hand area of the contralateral primary motor cortex (M1). Interestingly, the magnitude of this increase predicted long-term savings: Participants who showed greater gray matter increases in M1 after initial training with rotated feedback also showed a faster rate of adaptation to the same rotated feedback one year later. Recently, another study^17^ examined how changes in resting state functional connectivity were related to savings of adaptation. They found that participants showed increased connectivity in a motor network – including primary motor, premotor, posterior parietal cortex, cerebellum, and putamen – after performing a manual adaptation task. In addition, the extent of the connectivity increase within this network was associated with savings 24 h after initial adaptation. Remarkably, participants who showed more strengthening of the sensorimotor network demonstrated *less* savings. In another recent study^8^ transcranial direct current stimulation (tDCS) was employed to evaluate whether the right and left dorsolateral prefrontal cortex (DLPFC) and left M1 contribute to savings. Participants adapted dart-throwing movements while wearing laterally displacing prism lenses and while receiving anodal tDCS to one of the aforementioned sites. While stimulation did not modulate adaptation rates during the first test session, tDCS applied to the right DLPFC resulted in enhanced savings 48 h after initial adaptation. This suggests that the right DLPFC contributes to savings of adaptation.

Indirect indications of the neural correlates of savings of adaptation come from studies showing impaired savings in patients with Parkinson’s disease. Specifically, these patients showed impaired savings within a single test session^18^, 24 h after initial adaptation^7,19^, and 48 h after initial adaptation^20^. These behavioral results suggest that cortico-striatal pathways are involved in both within-session and multi-day savings.

To date, there have been no whole brain functional MRI (fMRI) studies of savings of adaptation. Here, we use fMRI to study the time course of activation over four sessions of sensorimotor adaptation administered over a three-month period. We examined activation during the first test session to evaluate which brain regions were involved in adaptation, distinguishing between early and late phases (i.e., first vs. second half of adaptation trials). In line with previous studies on the neural correlates of visuomotor adaptation, we hypothesized that activation would be greater during early adaptation than late adaptation in a variety of frontal and parietal areas including the DLPFC^1,4^, the cingulate cortex^2,21^, and the precuneus^2,21^. We further hypothesized that activation levels in these areas would correlate with individual differences in early adaptation rate (cf.^1,2^), thus serving as individual neural predictors of sensorimotor adaptability.

The novelty here is that we also studied whether and how the neural correlates of adaptation changed over the four test sessions. In addition, we examined individual neural predictors of savings across test sessions, by identifying areas in which activation levels correlated with individual differences in the extent of multi-day savings. The prospect of predicting at the individual level who will show most savings of adaptation could have important implications for training programs that facilitate astronaut adaptation to novel environments^22,23^ and rehabilitation^24^. We hypothesized that savings would show more neural overlap with early than with late adaptation, on the basis of recent literature linking savings to explicit recall of a previously successful adaptation strategy^14,15^. For example, the right DLPFC has been linked with working memory contributions to early adaptation^1,4^ and selection of goal-directed actions^25^. Indeed, recent findings suggest a role for the right DLPFC in savings^8^. Furthermore, previous studies have shown the anterior cingulate cortex (ACC) is recruited during early adaptation^21^ and plays a role in assigning value to actions on the basis of relevant feedback^26^. We hypothesized that these and other early-adaptation brain regions would be related to multi-day savings in sensorimotor adaptation. We interpret our findings in relation to a control experiment to account for kinematic differences in performance that were evident with savings, such as reduced error and faster movement times^27^. We have previously leveraged the findings from this control experiment to interpret findings related to transfer of adaptation learning^28^.

## Materials and Methods

### Participants

Sixteen healthy volunteers (12 male, 4 female) participated in this study. The same sample was included in a study on associations between adaptation learning and savings in manual and locomotor tasks^5^, in which behavioral data from the current task were also reported. Participants were aged 26 to 59 years (*M* = 40.8, *SD* = 8.7) at the time of the first test session and were recruited from the Test Subject Facility at the NASA Johnson Space Center. They reported having normal vision (five used reading glasses) and, with the exception of two participants, all were right-handed. They needed to pass standard eligibility criteria for MR imaging. Written informed consent was obtained from all participants and they received a compensation of $10 per hour for their participation. We excluded data from two participants from all analyses because of excessive head motion during the fMRI procedure (see fMRI data processing section below). Three other participants (including those two who were left-handed) were presented with a different experimental protocol after the first test session, and their data were therefore not included in the analyses that concerned all four sessions. Thus, the analyses for test session 1 were performed on data from 14 participants, whereas the analyses including subsequent sessions involved data from 11 participants. The study was conducted in accordance with the declaration of Helsinki, and was approved by the institutional review boards of the University of Michigan and NASA Johnson Space Center.

### Experimental task and procedure

**Figure 1:**
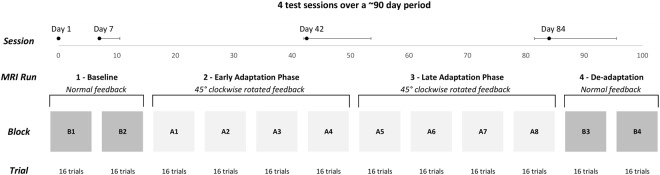
Overview of the study design. Participants completed the manual adaptation task while lying supine in the MRI scanner in four separate test sessions (median days and corresponding interquartile ranges for the test sessions are indicated in the top row). The task consisted of 40 s adaptation blocks, alternated with 20 s visual fixation periods.

Participants moved an MRI-compatible dual-axis joystick with the thumb and index finger of their right hand to hit targets presented on a screen (which they viewed via a mirror in the scanner), while lying supine in the MRI scanner. They received real-time feedback about the joystick location as a cursor on the screen, using a scaling factor of 1. Each movement was initiated from the central position on the display screen. Every 2.5 s a target appeared 4.8 cm to the right, to the left, above, or below the centrally located home position. Participants were instructed to move the cursor to the target as quickly as possible by using the joystick, and to hold the cursor within the target until it disappeared (1.5 s after presentation). They were further instructed to release the joystick handle after target disappearance, allowing the cursor to re-center to the home position. The next trial began 1 s later, resulting in an inter-stimulus interval of 2.5 s. As illustrated in Fig. 1, participants completed four fMRI runs of experimental trials (~13 minutes in total). The first run involved two blocks (B1 and B2) of 16 trials each under normal visual feedback. The next two runs consisted of four blocks of 16 trials each, with 45° clockwise rotated feedback (128 total trials; blocks A1–A8). Finally, the fourth run involved two blocks (B3 and B4) that were identical to those in the first run, allowing us to measure the aftereffects of adaptation. Each 40 s block was alternated with 20 s visual fixation periods. There was a longer interval between the successive runs – approximately 1–2 minutes – that was not strictly controlled. This blocked design was repeated on four different test dates, allowing us to examine adaptation learning and savings at subsequent test dates. Figure 1 (on the “Session” line) shows the median days (and interquartile ranges) after the first session at which the second, third and fourth test sessions were completed. As deadaptation under veridical feedback was successful at returning subjects’ performance to baseline level for the subsequent test session (indicated by the absence of a significant performance difference between the final five trials of blocks B2 and B4, *ps* > 0.09), the longitudinal design of the current study allowed us to use subjects as their own controls.

### fMRI acquisition parameters

Functional images were acquired on a 3 T Siemens Magnetom Verio MRI scanner located at the University of Texas Medical Branch at Victory Lakes, using a gradient echo T2*-weighted echo-planar imaging (EPI) sequence. The field of view (FOV) was 240 × 240 mm with a 94 × 94 matrix resulting in an in-plane voxel resolution of 2.55 × 2.55 mm. Repeat time to accomplish a full volume (TR) was 3660 ms, echo time (TE) was 39 ms, and the flip angle was 90°. Thirty-six axial slices of 4 mm thickness (with 1 mm slice gap) were collected in an interleaved multi-slice mode, covering the whole brain. Structural images were acquired using a T1-weighted gradient-echo pulse sequence (TR = 1900 ms, TE = 2.32 ms, flip angle = 9°) with a FOV of 250 × 250 mm and with a 512 × 512 matrix, resulting in an in-plane voxel resolution of 0.49 × 0.49 mm. We collected 192 slices with a slice thickness of 0.90 mm (scan duration ~6 minutes).

### Behavioral data processing and analyses

Performance was primarily assessed by measuring direction error (DE), defined as the angle between the line from the cursor’s origin to the target position and the line from the origin to the position at the time of peak velocity (cf.^1,2,4,29,30^). As several participants would occasionally attempt to guess where the target would appear and move the joystick in a (wrong) direction of their choice without attending to the target location, we replaced trials where the DE deviated more than 2.5 standard deviations from the mean across a session by the mean of the directly preceding and succeeding trial to minimize the influence of such trials (cf.^5,31^). This was done separately for each of the four sessions per participant and resulted in the replacement of 1.8% of the trials overall. To examine participants’ performance we ran a repeated measures analysis of variance (ANOVA) on DE with Session (4), Block (12), and Trial (16) as within-subject variables. As aforementioned, this analysis included the data from 11 of the 16 participants who completed an identical experimental protocol. The Huynh-Feldt correction was applied when the assumption of sphericity was not met and the threshold for significance was set at *p* < 0.05. Significant effects were further explored using post-hoc ANOVAs for each block to test for significant performance differences among sessions and/or trials.

Furthermore, we determined each participant’s rate of learning during the first test session by calculating the decay constant across adaptation trials (fit using an exponential decay function). This was used as the primary outcome measure for studying predictors of adaptability. We differentiated between the rate of learning during the early adaptation phase (i.e., run 2, trials in blocks A1–A4) and the late adaptation phase (i.e., run 3, trials in blocks A5–A8). We found that for both early and late adaptation the exponential decay function resulted in better fits than a linear function, *ts*(13) > 4.02, *ps* ≤ 0.001. In combination with previous findings that sensorimotor adaptation data can generally be characterized well by exponential decay functions^15,32,33^, this justifies our use of decay constants to quantify and analyze individual adaptation rates. Example single subject data and fitted functions are illustrated in Supplementary Fig. S1. Finally, to examine savings of adaptation we additionally calculated individual savings scores for test sessions 2, 3, and 4. Specifically, savings was defined as the average difference in DE across trials in block A1 (i.e., first 16 trials with rotated feedback) between a test session and its preceding session. Importantly, learning rates and savings scores were calculated on an individual basis for each participant.

### fMRI data processing

Statistical Parametric Mapping software version 8 (SPM8; Wellcome Trust Center for Neuroimaging) running in the Matlab environment (Mathworks, Sherborn, MA, USA) was used for slice timing and motion correction. Slice timing correction to the first slice was performed using SPM’s sinc interpolation. Head motion correction was performed by co-registering each image to the mean EPI image. To examine outliers due to spiking and motion, and additionally to estimate Euclidian motion, we used the Artifact Detection Tool software package (ART)^34^. The amount of translation and rotation about each of the axes was examined and as two participants showed head motion greater than 3 mm during the experiment, their data were excluded from further analyses.

The fMRI data were normalized using Advanced Normalization Tools software (ANTs)^35^ after a multi-step approach in which we (1) pre-processed the T1-weighted image; (2) calculated the warp parameters from the T1-weighted image to an MNI152 template; and (3) applied these warp parameters to the fMRI data. First, for pre-processing of the structural data, the in-plane resolution was down sampled to 0.94 × 0.94 mm. Image intensity non-uniformity correction was estimated and applied to all T1 images within a subject-specific brain mask using N4ITK^36^. The brain masks were created using FSL’s Brain Extraction Tool (BET)^37^ with robust brain center estimation and a fractional intensity threshold of 0.2. For each participant we then co-registered the structural pre-processed T1-weighted image to the mean functional image, after which we spatially normalized the obtained images to the MNI template^38^. The warp from the single subject T1 to the MNI152 template was calculated using ANTs with cross correlation as the similarity metric and symmetric normalization as the transformation model^35^. Finally, the resulting normalization parameters were applied to the participant’s functional images, which were then spatially smoothed with a Gaussian kernel with a sigma of 4 mm (i.e., ~9.4 mm FWHM) using FMRIB Software Library (FSL)^39^.

To obtain the best normalization accuracy for the cerebellum, we isolated the cerebellum using a spatially unbiased atlas template of the cerebellum and brainstem (SUIT)^40^ and then registered the isolated cerebellum to the MNI152 cerebellum that was normalized to the SUIT template. The remaining cerebellar normalization procedure was identical to that for the whole brain.

### fMRI data analyses

The fMRI data analyses were performed with SPM12. We used general linear models combined with an estimate of the hemodynamic response function for statistical analysis. High-pass filtering at 128 s was used to remove low frequency drift. Head motion parameters were included as covariates of no interest in our model to rule out potential confounding effects induced by head movement. In addition, outliers related to spiking and motion as detected by the ART toolbox were regressed out from the signal. We first performed analyses at the single-subject level and subsequently carried the resulting contrast images over to our group analyses.

We ran one-sample t-tests to evaluate which areas are involved with the early and late phases of sensorimotor adaptation. The first contrast examined the difference between activation during adaptation blocks (runs 2 and 3) and activation during baseline performance (run 1). Another contrast examined activation differences between the early versus late adaptation phases (run 2 vs. run 3). Moreover, we ran two multiple regression models in which participants’ individual learning rates were entered as a covariate. This was done to search for brain regions in which activation was correlated with the rate of learning during early and late adaptation phases, respectively. These analyses were performed only on the data from the first test session and included the data of 14 participants.

We also evaluated areas showing changes in activation over the four test sessions. Using a linear mixed model (implemented under “flexible factorial design” in SPM), we searched for brain regions that exhibited increasing or decreasing activation over the test sessions. Finally, to examine regions associated with multi-day savings, we used a flexible factorial design in which savings scores for test sessions 2, 3, and 4 were included as covariates. These latter analyses included the data from 11 participants. We performed each of these analyses on both the whole brain and the cerebellar images. For the whole-brain analyses, we masked out the cerebellum, and for the cerebellar analyses we masked out cortical areas. All effects were evaluated using a statistical threshold of *p* < 0.0005 (uncorrected for multiple comparisons) and a minimum cluster size of 10 voxels; a few effects were significant at a family-wise error (FWE) corrected *p* < 0.05, as indicated in the tables and text. We used the Harvard-Oxford Cortical and Subcortical Structural Atlases^41^ for localization of cortical and subcortical areas, and the probabilistic cerebellar atlas^42^ for localization of cerebellar areas.

## Results

### Behavioral results

**Figure 2:**
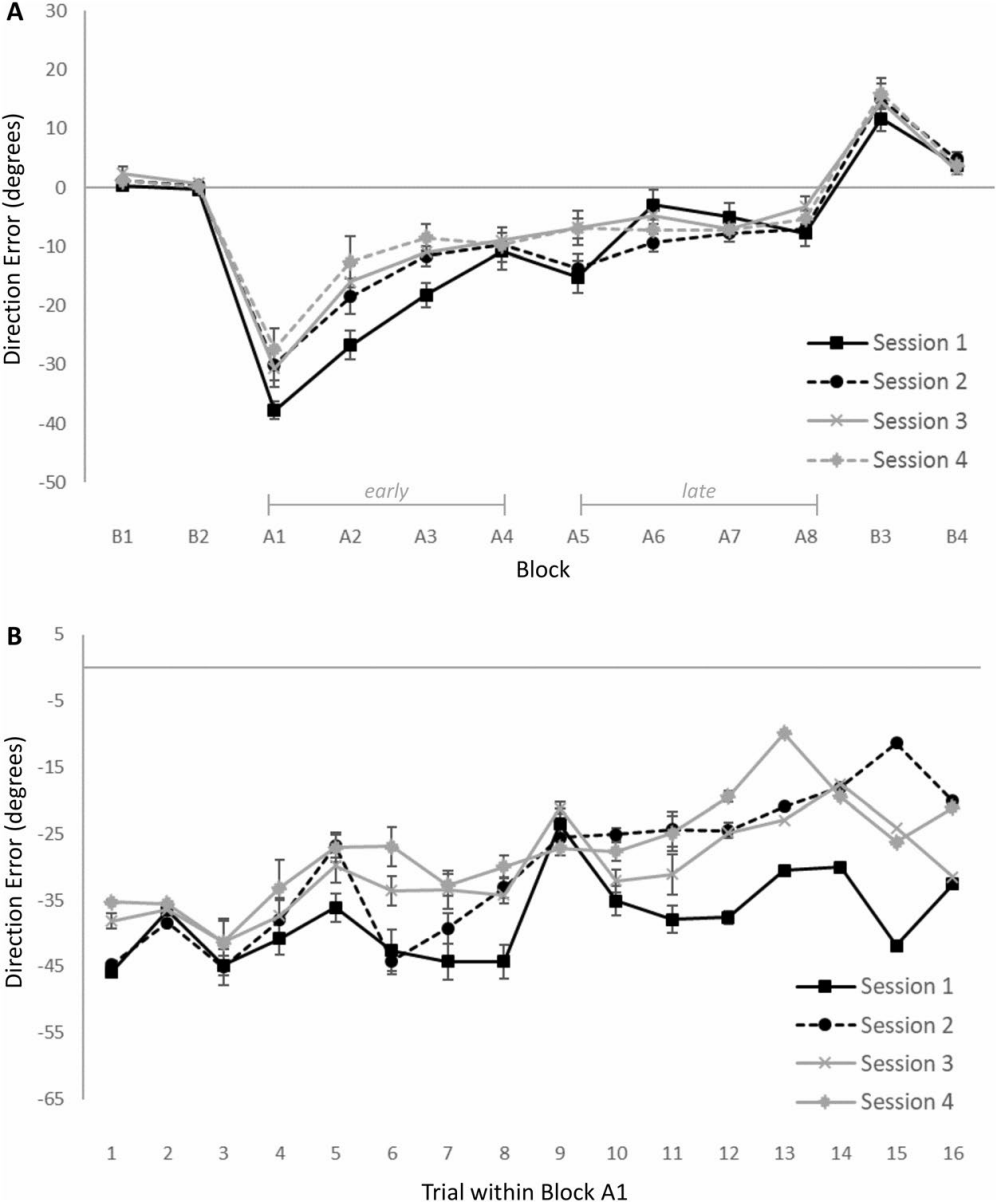
(**A**) Mean DE for blocks in the adaptation task as a function of test session. Error bars represent standard errors. (**B**) Mean DE for trials in the test block where the rotated feedback was first introduced (i.e., A1) as a function of test session. N.B.: While DEs in these figures are averaged across all participants, it should be noted that learning rates and savings scores were determined on an individual basis.

Results of the Session × Block × Trial repeated measures ANOVA on DE showed a main effect of Block, *F*(11, 110) = 70.24, *p* < 0.001, *η*_*p*_^2^ = 0.87, as well as a Block × Trial interaction, *F*(165, 1650) = 2.70, *p* < 0.001, *η*_*p*_^2^ = 0.21. Follow-up analyses showed no indications that performance changed across trials in baseline blocks B1 and B2 (*ps* > 0.40). In contrast, DEs decreased across the trials in blocks A1–B4, *Fs* > 1.80, *ps* < 0.05, *η*_*p*_^2^*s* > 0.15. These performance changes reflect within-block improvements after the initial drop in performance to the rotated feedback in blocks A1–A8. In addition, the “overshooting” of the baseline target upon removal of the rotated feedback gradually reduced in blocks B3 and B4, reflecting deadaptation once the rotated feedback was removed. Interestingly, results also showed a Session × Block interaction, *F*(33, 330) = 3.29, *p* < 0.001, *η*_*p*_^2^ = 0.24. Follow-up contrasts indicated that DE changed significantly across the sessions in blocks A1, A2, A3, A5 and A6, *Fs* > 4.42, *ps* < 0.05, *η*_*p*_^2^*s* > 0.31. As illustrated in Fig. 2A, this suggests that savings occurred across the four test sessions in these blocks.

To verify that participants showed savings of previous learning, we determined individual savings scores for test sessions 2–4. The difference between each participant’s mean DE on trials in the first adaptation block (i.e., A1; the first 16 trials with rotated feedback) during a test session and the mean DE in that block during the preceding session was taken as an indicator of savings. Subjects’ individual savings scores ranged from −33.99 to 4.08 degrees on test session 2, from −12.81 to 9.28 degrees on test session 3, and from −12.84 to 3.96 degrees on test session 4 (where more negative scores reflect more savings). We ran one-sample t-tests (one-tailed) on mean savings scores across all participants in each of the test sessions to evaluate whether they were smaller than zero. In test sessions 2 and 4, participants showed significant savings relative to the previous session (*M* = −7.81, *SE* = 2.92 and *M* = −3.23, *SE* = 1.65, respectively), *ts*(10) < −1.96, *ps* < 0.039, but not in session 3 (*M* = 0.62, *SE* = 1.82, *p* = 0.74).

We also evaluated savings occurring within the first block itself. To achieve this, we performed linear contrasts to evaluate whether changes in DE across trials in block A1 were different between successive sessions. Results revealed that DE improved significantly faster across trials in session 2 compared to session 1, *F* = 5.83, *p* = 0.036, *η*_*p*_^2^ = 0.37 (see Fig. 2B). A similar trend was observed for the comparison between sessions 2 and 3, *F* = 4.52, *p* = 0.060, *η*_*p*_^2^ = 0.31, but not sessions 3 and 4 (*p* = 0.67). To evaluate whether there were any session differences in DE at the start of block A1, we determined average DE across the first few adaptation trials (cf.^43,44^). Results of a repeated measures ANOVA on the mean DE across the first three trials with Session (4) as within-subject variable showed that DE did not differ significantly across the sessions (*p* = 0.46). These results further support the notion that the session difference in A1 mean DE reflects savings and suggest that such savings may be related to explicit awareness of the perturbation (cf.^15^).

### Functional imaging results

We observed greater activation for adaptation (blocks A1–A8) than baseline performance (blocks B1 and B2) in the left angular gyrus. Results additionally showed that activation was greater for early than for late adaptation in the left cerebellum (lobules I–IV, crus II, and lobule VIIb). In contrast, activation was greater for late than for early adaptation in the left superior frontal gyrus and left central operculum. These results are summarized in Table 1 (contrasts 1–3). No other results were obtained at a significance level of *p* < 0.0005.

**Table 1:** Overview of the results for contrasts performed on test session 1 (*n* = 14).

Contrast	Anatomic location	Brodmann’s area	Coordinates of peak	Cluster size (voxels)	*Z* score
*1. Adaptation* > *Baseline*					
	L angular gyrus	39	−36, −56, 36	11	3.75
*2. Early* > *Late*					
	L CB crus II		−8, −78, −42	82	3.55
	L CB lobules I-IV		−3, −55, 1	32	3.54
	L CB lobule VIIb		−18, −70, −48	19	3.44
*3. Late* > *Early*					
	L SFG	8	−16, 20, 44	11	4.03
	L central operculum	13	−42, −12, 18	22	3.72
*4. Early adaptation correlation*					
	**Frontal**				
	R MFG (DLPFC)	9	44, 14, 36	21	3.85
	L IFG (DLPFC)	46	−40, 28, 12	56	4.15
	**Cingulate**				
	L dACC	32	−14, 20, 40	16	3.98
	L pCG	31	−2, −26, 36	23	3.67
	**Temporal**				
	L MTG	37	−48, −62, 2	24	3.91
	**Subcortical**				
	L thalamus		−2, −12, −2/−2, −10, −6	23	4.11/3.38
	L putamen		−26, 6, 0/−28, 2, −4	11	3.49/3.34
*5. Late adaptation correlation*					
	**Frontal**				
	L MFG (dPMC)	6/8	−32, 8, 44/−36, 16, 48	64	4.44/3.80
	L PCG	10	−8, 48, 6	11	3.59
	**Temporal**				
	L STG	22	−52, −36, 18	12	3.56
	**Cingulate**				
	R dACC	32	4, 28, 30	10	3.51
	**Occipital**				
	R cuneus	18/19	4, −80, 36/16, −90, 32	97	4.19/4.09
	L CALC (V1)	17	−16, −70, 12/−14, −66, 14	82	3.90/3.80

(1) Region engaged more in adaptation than in baseline performance. (2) Regions engaged more in early than late adaptation. (3) Regions engaged more in late than early adaptation. (4 and 5) Regions that show a correlation across participants between activation and the rate of learning within the early and late adaptation phases, respectively. Note that greater activation was associated with *faster* adaptation during the early adaptation phase, but with *slower* adaptation during the late adaptation phase. CB = cerebellum; SFG = superior frontal gyrus; MFG = middle frontal gyrus; DLPFC = dorsolateral prefrontal cortex; IFG = inferior frontal gyrus; dACC = dorsal anterior cingulate cortex; pCG = posterior cingulate gyrus; MTG = middle temporal gyrus; PCG = paracingulate gyrus; dPMC = dorsal premotor cortex; STG = superior temporal gyrus; CALC = calcarine cortex; V1 = primary visual cortex.

**Figure 3:**
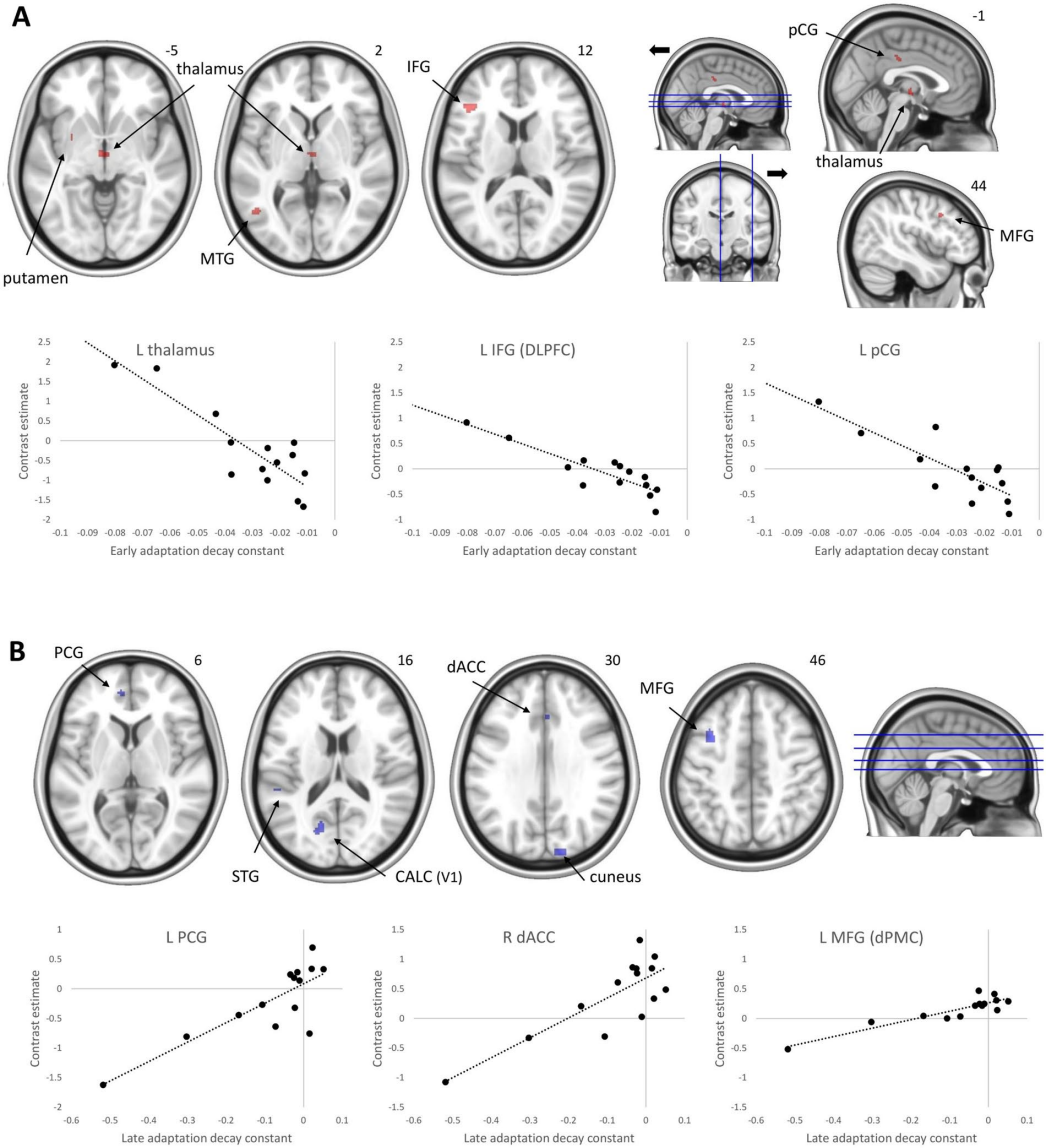
Areas showing associations with adaptation rate for the early adaptation phase (panel A; activation associated with faster adaptation) and the late adaptation phase for the group of subjects (panel B; activation associated with slower adaptation). Scatter plots illustrate the association between adaptation rate and activation for selected areas; note that more negative values reflect faster learning. The right side of each image corresponds to the subjects’ right side. MTG = middle temporal gyrus; IFG = inferior frontal gyrus; pCG = posterior cingulate gyrus; MFG = middle frontal gyrus; PCG = paracingulate gyrus; STG = superior temporal gyrus; CALC = calcarine cortex.

The DE exponential decay constant across trials in the first four adaptation blocks (A1–A4) and the decay constant in the subsequent four adaptation blocks (A5–A8) in test session 1 were taken as indicators of adaptation rate during the early and late phases, respectively. Subjects’ individual decay constants in the first test session ranged from −0.08 to −0.01 for the early adaptation phase and from −0.52 to 0.05 for the late adaptation phase, with more negative values reflecting faster learning. Table 1 (contrasts 4 and 5) presents the brain regions showing a correlation across participants with individual differences in the rate of learning during early and late adaptation in test session 1. For the early adaptation phase, greater activation in the presented regions was associated with *faster* adaptation (larger negative decay constants). These regions (Fig. 3A) encompassed a variety of frontal, temporal, and cingulate cortical and subcortical areas, including the bilateral DLPFC, left dorsal ACC (dACC), and left putamen. We did not observe any brain regions in which greater activation was associated with slower adaptation in the early phase. For the late adaptation phase, greater activation in the presented regions was associated with *slower* adaptation (smaller negative decay constants). These regions (Fig. 3B) included a variety of frontal, cingulate, and occipital cortical areas. There were no brain regions in which greater activation was associated with faster adaptation in the late phase. As visual inspection of the scatter plots in Fig. 3A,B suggested that the observed associations could potentially be driven by extreme data points, we tested the data for outliers. Grubbs’ test indicated that the lower-left data point in the scatter plots of Fig. 3B was an outlier. We therefore excluded this data point and re-ran the correlation analyses. Results showed that with exception of the STG, activation in the presented regions remained to be significantly associated with faster adaptation. No outliers were detected for the data presented in the scatter plots of Fig. 3A.

**Figure 4:**
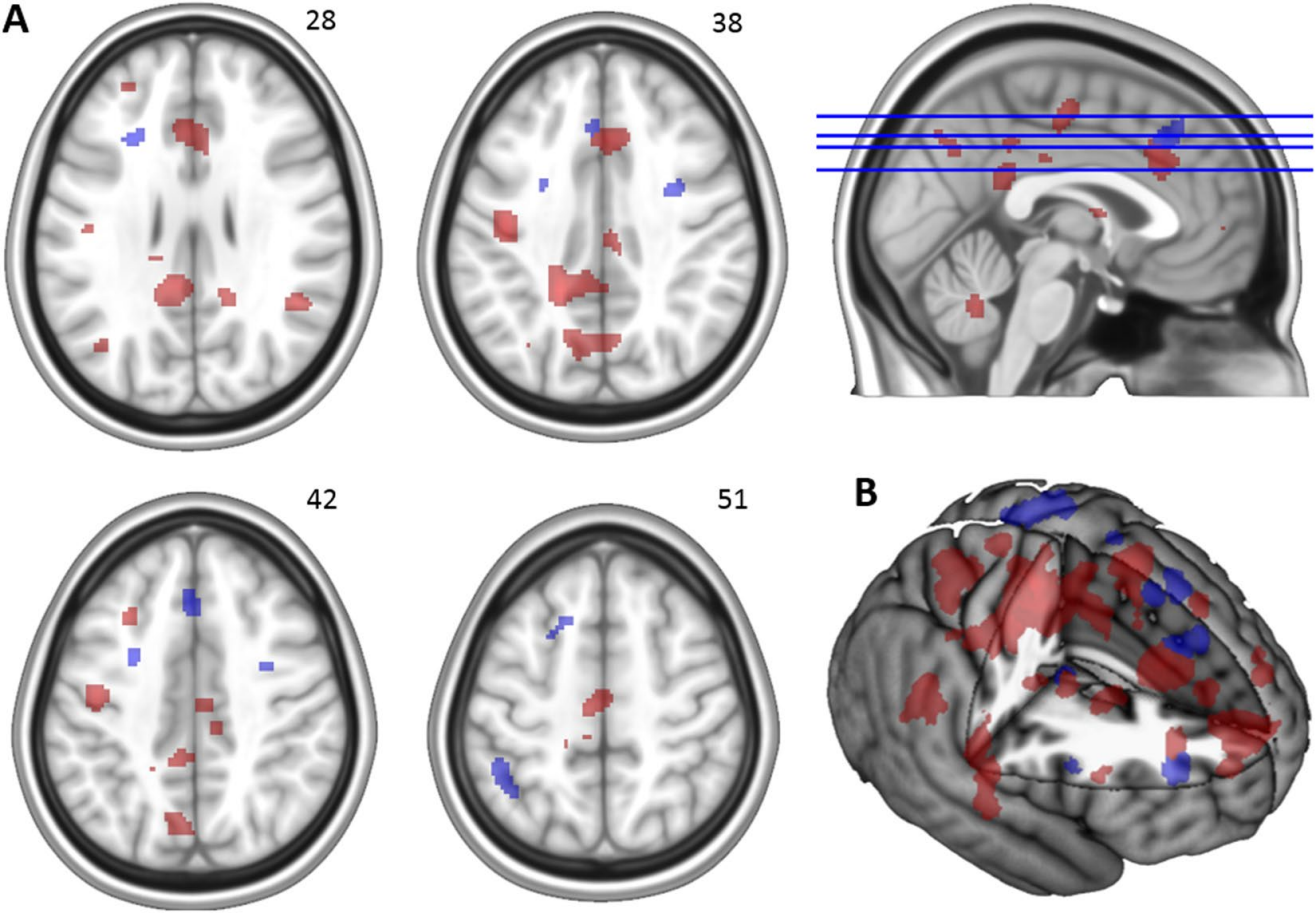
Panel A: Selected areas showing activation changes over the four test sessions. For the early adaptation phase, such changes mainly involved increases (red), while for the late adaptation phase results mainly showed decreases (blue). Panel B: Rendered profile of brain areas showing changes in activation over the four test sessions. The right side of each image corresponds to the subjects’ right side.

Regions showing changes in activation over the four test sessions of the present study are shown in Table 2 and Fig. 4. For the early adaptation phase (blocks A1–A4), results revealed increased activation over time in bilateral dACC that was significant at an FWE-corrected threshold. At the conservative uncorrected threshold, activation increased over the sessions in a variety of frontal, parietal, and cingulate cortical areas, as well as various subcortical areas. In contrast, activation in this phase decreased over time in the left cerebellum (crus II). For the late adaptation phase (blocks A5–A8), activation increased over time in the left thalamus, while activation decreased over time in a variety of frontal and parietal cortical areas.

**Table 2:** Regions that show activation changes across participants (*n* = 11) over the four test sessions during the early and late adaptation phases, respectively.

Phase	Anatomic location	Brodmann’s area	Coordinates of peak	Cluster size (voxels)	*Z* score
*Early adaptation*					
	**Frontal**				
	L MFG (dPMC)	8/10	−28, 22, 42/−34, 44, 16/	38/56	4.25/4.07/
			−28, 46, 30/−26, 52, −2	16/350	4.00/4.37
	L SMA	6	−6, −14, 56	988	4.27
	L PCG	32	−14, 48, 0	350	4.57
	L preCG	4/6	−42, −14, 40/−6, −20,50	240/988	4.70/4.06
	L postCG	5	−16, −34, 54	27	3.46
	R PCG	10	4, 54, 0	39	3.75
	R postCG	5	14, −32, 56	27	3.63
	L operculum	4	−42, −14, 40/−46, −18, 24	240	4.70/4.23
	**Parietal**				
	L precuneus	7	−10, −68, 38	256	3.87
	L operculum	13	−32, −26, 22	79	4.13
	R precuneus	31	2, −66, 36	256	3.82
	R angular gyrus	39/40	46, −50, 28/ 58, −52, 22	127	4.26/3.46
	**Cingulate**				
	L dACC	32	−12, 34, 18	34	3.77
	L pCG*	31	−16, −42, 34	988	5.12
	R rACC	32	10, 32, 8	47	4.02
	R dACC *	8	2, 22, 34	335	4.81
	R pCG	23/31	16, −46, 30/10, −28, 42	51/988	4.32/3.67
	**Temporal**				
	L PT	41	−48, −28, 4	16	3.55
	R PT	41	54, −20, 4	111	4.7
	R STG	22	60, −16, −6	111	4.14
	**Occipital**				
	L OFG	18 /19	−24, −80, −4/−32, −64, −4	12/34	3.50/3.76
	L LOC	39/19	−36, −66, 32/−44, −62, 18	71/60	4.00/3.79
	**Subcortical**				
	L thalamus		−8, −2, 6	70	3.84
	L hippocampus		−20, −30, −8	36	3.68
	R thalamus		4, −2, 10	70	3.67
	R putamen		26, 12, −2	15	3.48
	**Cerebellum**				
	Vermis VIIIa*		−5, −57, −30	1183	4.7
	L Lobule V		−23, −46, −15/−21, −42, −15	109	3.67/3.53
	L Crus I		−8, −82, −19	6	3.39
	L Crus II^↓^		−32, −60, −44	12	3.45
*Late adaptation*					
	**Frontal**				
	L MFG	9/6/8	−26, 22, 30/−26, 4, 42	49/34	4.33/4.13
	L PCG	8	−2, 28, 42	85	4.19
	L SFG	6	−16, 20, 54/−22, 12, 54	74	4.13/3.62
	R MFG	8/10	32, 0, 40/28, 42, 12	39/71	4.17/4.05
	R IFG	44	56, 16, 20	12	3.49
	**Parietal**				
	L SMG	40	−44, −48, 54/−62, −34, 32	152/11	3.97/3.50
	L angular gyrus	39/40	−38, −56, 48/−42, −52, 52	152	3.92/3.75
	**Subcortical**				
	L thalamus^↑^		0, −16, 2	36	4.16

All regions listed for the early adaptation phase showed *increases* in activation, with the exception of L cerebellum crus II. All regions listed for the late adaptation phase showed *decreases* in activation, except for the left thalamus. MFG = middle frontal gyrus; SMA = supplementary motor area; PCG = paracingulate gyrus; preCG = precentral gyrus; postCG = postcentral gyrus; dACC = dorsal anterior cingulate cortex; pCG = posterior cingulate gyrus; rACC = rostral anterior cingulate cortex; PT = planum temporale; STG = superior temporal gyrus; OFG = occipital fusiform gyrus; dPMC = dorsal premotor cortex; LOC = lateral occipital cortex; SFG = superior frontal gyrus; IFG = inferior frontal gyrus; SMG = supramarginal gyrus.

*=remained significant at FWE-corrected *p* < 0.05.

**Figure 5:**
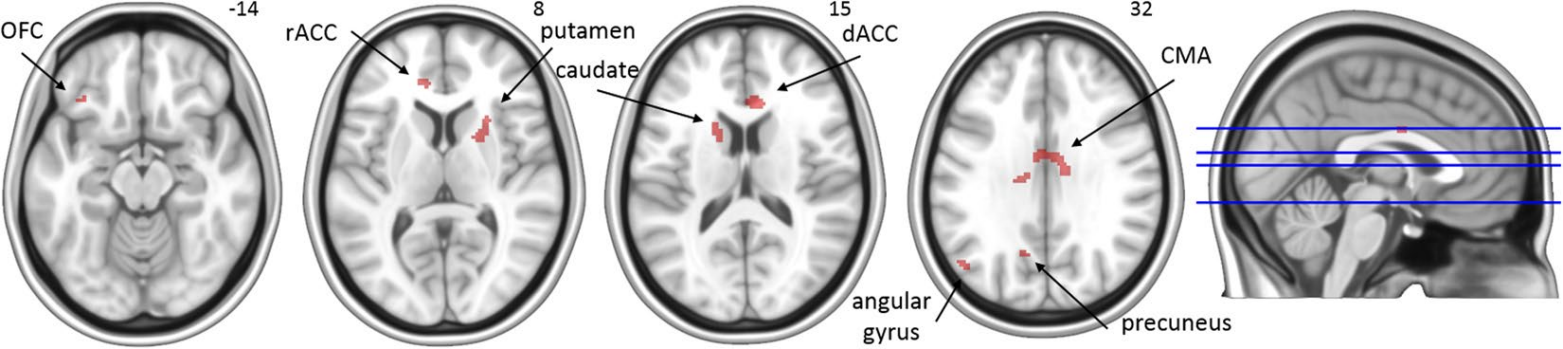
Areas in which activation during the early adaptation phase was associated with more savings. The right side of each image corresponds to the subjects’ right side. OFC = orbitofrontal cortex; rACC = rostral anterior cingulate cortex; dACC = dorsal anterior cingulate cortex; CMA = cingulate motor area.

**Figure 6:**
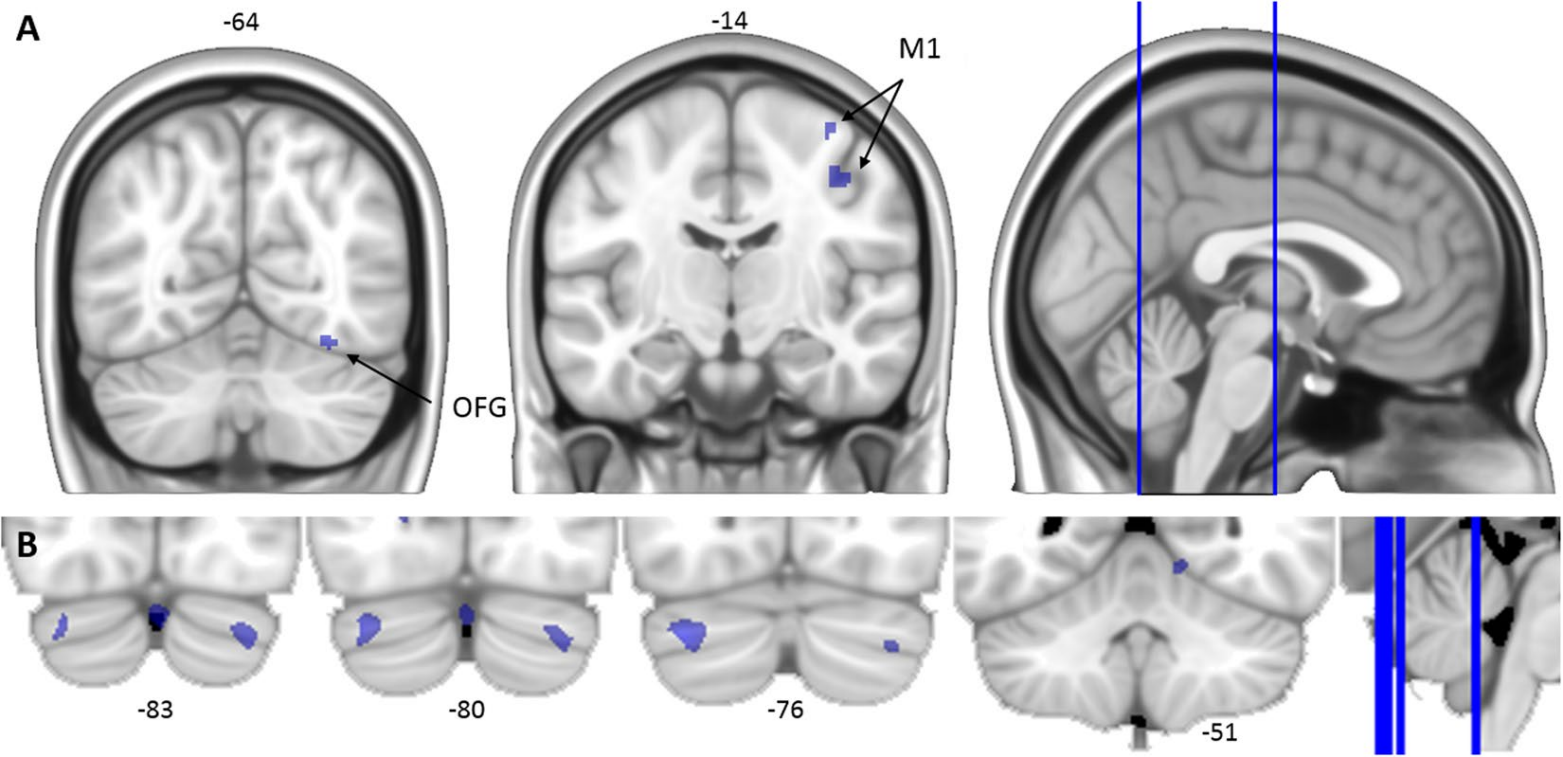
Cortical (panel A) and cerebellar (panel B) areas in which activation during the early adaptation phase was associated with less savings. The right side of each image corresponds to the subjects’ right side. OFG = occipital fusiform gyrus; M1 = primary motor area.

Table 3 presents the regions in which task-related activation showed a correlation across participants and across test sessions 2, 3, and 4 with individual differences in savings score. Participants who showed more savings of what they learned during the previous test sessions demonstrated greater activation in a variety of frontal, parietal, and subcortical areas during the early adaptation phase. Figure 5 shows that these included the bilateral dorsal anterior cingulate and cingulate motor areas, left precuneus, and left angular gyrus. In contrast, results showed that participants who showed less savings demonstrated greater activation during the early adaptation phase in the left cerebellum (crus I); this correlation was significant at an FWE-corrected threshold. At the uncorrected threshold, the same association was additionally observed for activation in a variety of cerebellar areas and motor cortical areas, including the right primary motor cortex and the supplementary motor area (Fig. 6). More savings of adaptation learning was also associated with greater activation in the left superior frontal gyrus during the late adaptation phase, while less savings was associated with greater activation in the right middle occipital gyrus and cerebellum (lobule VI) during this phase.

**Table 3:** Regions that show a correlation across participants (*n* = 11) and test sessions between activation and savings score.

Phase	Anatomic location	Brodmann’s area	Direction	Coordinates of peak	Cluster size (voxels)	*Z* score
*Early adaptation*						
	**Frontal**					
	L OFC	47	+	−36, 28, −14	11	3.81
	R preCG (M1)	4/6	−	38, −16, 44/38, −14, 64	42	3.79/3.51
	**Parietal**					
	L precuneus	31	+	−12, −64, 28	13	3.67
	L angular gyrus	39	+	−50, −70, 30	24	3.97
	**Occipital**					
	R OFG	19	−	32, −64, −16	19	3.88
	**Cingulate**					
	L CMA	24	+	−2, −4, 30	69	3.95
	L rACC	32	+	−12, 38, 6	19	3.68
	R CMA	24	+	4, −6, 30	69	3.87
	R dACC	24	+	8, 26, 16	74	4.39
	**Subcortical**					
	R putamen		+	18, 8, 4/26, 16, 8	75	4.14/3.62
	L caudate		+	−16, 10, 16	59	3.91
	**Cerebellum**					
	L Crus I*		−	−34, −76, −33/−33, −81, −29	758	4.89/4.41
	R Crus I		−	31, −83, −33	446	4.18
	R Crus II		−	5, −83, −26	47	3.43
	Vermis, crus II		−	1, −80, −27	47	3.84
	R Lobule V		−	14, −51, −10	73	3.68
	R Lobule VIIb		+	38, −55, −48	10	3.46
*Late adaptation*						
	**Frontal**					
	L SFG	6	+	−10, 12, 63	22	3.85
	**Occipital**					
	R MOG	19	−	36, −74, 8	17	4.06
	**Subcortical**					
	Cerebellum					
	L Lobule VI		−	−16, −62, −27	175	3.79

In the Direction column, +denotes that more savings was associated with greater activation, whereas – denotes that less savings was associated with greater activation. OFC = orbitofrontal cortex; preCG = precentral gyrus; M1 = primary motor area; OFG = occipital fusiform gyrus; CMA = cingulate motor area; rACC = rostral anterior cingulate cortex; dACC = dorsal anterior cingulate cortex; SFG = superior frontal gyrus; MOG = middle occipital gyrus.

*=remained significant at FWE-corrected *p* < 0.05.

## Discussion

In the present study we evaluated changes in neural activation that occurred over the time course of multiple days of sensorimotor adaptation, and identified individual neural predictors of adaptation and savings magnitude. We collected functional MRI data while participants performed a manual adaptation task during four separate test sessions over a three-month period. Behavioral results showed that participants’ performance under rotated feedback improved within each test session, thus reflecting adaptation. In addition, participants were less perturbed by the rotated feedback in later sessions than in the initial test session, indicating that savings of adaptation occurred. Neuroimaging results showed that brain activity associated with early adaptation increased across the four test sessions in a variety of frontal, parietal, cingulate, and temporal cortical areas, as well as various subcortical areas. We found that savings was positively associated with the level of activation in several striatal, parietal, and cingulate cortical areas including the putamen, precuneus, angular gyrus, dACC, and cingulate motor area. Below, we first briefly discuss our findings regarding the neural correlates of early and late adaptation during the initial test session. We then focus in more detail on our findings regarding the neural correlates of savings and their implications for the mechanisms underlying savings.

It is well known that brain activity changes with movement rate^45,46^ and error magnitude^47^ in various regions, which can potentially confound interpretations about brain changes occurring with adaptation and savings. Here we address this problem by interpreting our results in relation to a control experiment that we have performed^27^. Participants moved the same joystick device as in the current study to hit targets of varying sizes, inducing kinematic differences across conditions such as changes in peak velocity, movement time, and error magnitude, in the absence of adaptation. Thus, this data set elucidates brain regions that change their activity with performance in the absence of learning.

We examined regions showing activation specifically during adaptation, as well as activation differences between early and late adaptation phases. Results showed that activation was greater during adaptation than during baseline performance in the left angular gyrus. In addition, activation was greater for early adaptation than for late adaptation in various cerebellar areas, but greater for late adaptation than for early adaptation in the left superior frontal gyrus and left central operculum. While these observations are largely in line with earlier work^1,2,4,21^, we observed fewer areas that showed phase-specific activation than have been reported previously. This might be attributed to differences in definitions of early and late adaptation phases between the current and previous studies. That is, in the current study the early and late adaptation phases comprised the first and final 64 adaptation trials, respectively (out of 128 trials in total). In previous studies, however, these phases were defined as the first and final 24 adaptation trials of the experiment (out of 264 trials in total^21^; out of 72 in total^2^) or first and final 72 adaptation trials (out of 264 in total)^1,4^. These differences are a result of the early and late adaptation phases typically being defined within the context of the specific experimental design, considering for example the number of adaptation trials or successive training days. Possibly the current design may have yielded relatively smaller differences between early and late adaptation that were not detected using our current threshold of *p* < 0.0005. Although a more liberal threshold might have revealed such differences, it would have also increased the chances of finding false positives.

We identified several neural predictors of adaptability; greater activation in the bilateral DLPFC, left dACC, and left putamen was associated with faster adaptation during the early phase. This corresponds with earlier work showing that activation in areas related to cognitive processes, such as visuospatial working memory, is associated with adaptability^1,4^. In contrast, greater activation in areas that perform cognitive processing such as the dPMC and dACC during the late adaptation phase was associated with slower adaptation. The opposite relationship between the rate of adaptation during the early and late phases of adaptation and activation levels in cognitive processing areas suggests that people who are still engaging cognitive areas later in the task are doing more poorly and may need more time and/or practice to transition to more automatic performance. This interpretation would fit prior findings that faster rates of adaptation during the late phase are typically associated with greater activation in motor areas such as the sensorimotor cortex, cingulate motor area, and cerebellum^1,10^, although we did not observe such associations in the late phase in the current study.

To examine the neural correlates of savings, we evaluated changes in neural activation that occurred over the time course of four sessions of manual sensorimotor adaptation during a three-month period. In addition, we aimed to identify individual neural predictors of savings in such adaptation. Results showed differential activation changes over the four test sessions for the early and late phases of adaptation. Specifically, we observed that activation during the early adaptation phase increased in a variety of frontal, parietal, and cingulate areas. These increases could reflect that participants were facilitating cognitive processes of adaptation that benefited explicit adaptation strategies^6^, such that the reported areas were trained to increasingly contribute to adaptation to the rotated feedback upon re-exposure. A caveat of this interpretation is that our findings leave open the question of whether this involves implicit and/or explicit cognitive processes. Regardless, the observed increases in activation are unlikely to be linked to performance differences occurring across the test sessions; we have previously shown increasing anterior cingulate cortex activity in association with larger motor errors^27^, but here error magnitude is decreasing across sessions.

In contrast, activation in the late adaptation phase decreased over the test sessions in a variety of frontal and parietal cortical areas. These decreases potentially suggest that the development of changes in motor representations required to successfully cope with the perturbation may be accelerated with each subsequent exposure. As fronto-parietal networks have previously been shown to contribute to making online movement corrections in goal-directed motor performance^48^, reduced activation in these areas over the four test sessions may reflect more effective performance as learning advances.

We further identified areas in which activation was associated with adaptation savings. We observed that activation in predominantly motor areas that are typically involved in motor execution (M1 and various cerebellar regions) was associated with less savings, indicating that individuals who recruit these motor areas more during task performance show weaker savings of previous learning. This is in line with prior findings that greater strengthening of resting state functional connectivity in a motor network was related to weaker savings^17^. This association with motor execution areas is unlikely to be simply a performance effect; we have previously found motor cortical activity to be greater when participants move the joystick at a faster speed in a control experiment^27^, whereas in this case higher motor cortex activity is associated with *less* savings (and hence slower, higher error movements). While we believe that these opposite findings between the present study and the control experiment argue in favor of a role for savings rather than for performance differences, we acknowledge that we cannot conclusively refute the possibility that differences in motor performance across the four sessions contributed to the present results. For example, less savings may alternatively be linked to a need for more online corrections or to less efficient activation in motor areas.

We observed that activation in brain areas known to serve cognitive functions was associated with more savings. These areas encompassed frontal, parietal, and cingulate areas including the bilateral dACC and CMA, which have been previously reported to be involved in the early phase of adaptation^2,21^. These regions are also unlikely to reflect simple performance effects in the current experiment as we have previously shown increasing CMA activity for slower movements with larger errors in a control experiment^27^. Our results are in line with our hypothesis and corroborate the notion that savings of sensorimotor adaptation rely on mechanisms that overlap with those of early adaptation (cf.^14,15^). These likely include cognitive processes, such as visuospatial working memory^1,4,5^, that benefit the development and use of explicit adaptation strategies^6^. Notably, our findings regarding the areas related to savings were based on brain-behavior associations across all sessions after initial adaptation learning, thus making the present experimental design more powerful than a two-session study.

We propose that three different loops may be involved in multi-day savings in sensorimotor adaptation. One is a cortico-striatal loop including the dACC, CMA, putamen, thalamus, and caudate. The dACC is known to be involved in evaluating and assigning values to actions based on external feedback^26,49^. In sensorimotor adaptation, this loop may be involved in resolving the selection of competing movements (i.e., typical movements vs. those that are correct for counteracting the perturbation). Given the extensive dopaminergic projections of the dACC and CMA, the current finding that activation in cingulate areas was associated with multi-day savings is in line with previous studies showing impaired long-term savings of adaptation in patients with Parkinson’s disease^19,20^, as well as findings that activation in the ACC and the posterior putamen correlated with immediate (i.e., same day) recall efficacy^7^. A second loop is a fronto-parietal loop, which includes the OFC, precuneus, and angular gyrus. The former is involved in detecting and encoding the reward value of actions^50^, while the latter areas have been shown to be involved in visuospatial working memory^51^. It could therefore be argued that this loop is probably most directly related to the explicit mechanism underlying savings, and may be involved in improvements in action selection through strategic re-aiming or recall of a previously successful adaptation strategy^14,15^. The OFC detects and encodes the success of each performed action, which may then be evaluated by the cingulate cortico-striatal loop. As higher value is assigned to correct movements than to incorrect movements, the former are more likely to be performed again. Finally, a motor loop including M1 and the cerebellum may drive the actual execution and coordination of movements in the sensorimotor adaptation task. Given our observation that activation in these areas was associated with less savings, it seems that when individuals are less able to use explicit strategies to deal with the rotated feedback, the more they have to recruit the areas in this loop for appropriate motor performance.

In summary, the present study demonstrated neural changes across four different sessions of sensorimotor adaptation learning. Moreover, this study examined for the first time the neural mechanisms underlying multi-day savings over a three-month period in such adaptation using fMRI. We observed that more savings was associated with greater activation in various brain cognitive areas that are also known to be involved in early adaptation, whereas less savings was associated with greater activation in predominantly motor areas. Overall, these findings suggest that participants may be learning how to better engage cognitive processes across days, potentially reflecting the improvements in action selection that have been shown to occur with savings of adaptation. We propose that such improvements may rely on the evaluation of previous actions by way of a cortico-striatal loop involving the dACC, which assigns higher value to correct movements than to incorrect movements so that the former are more likely to be performed again.

## Electronic supplementary material

Supplementary Material

## Data Availability

The data that support the findings of this study are available on request from the corresponding author [R.S.].
