# Supplementary material for: Neural correlates of multi-day learning and savings in sensorimotor adaptation
Source: Sci Rep. 2018 Sep 24;8:14286. doi: 10.1038/s41598-018-32689-4 (PMC6155344; doi:10.1038/s41598-018-32689-4)

Supplementary Material

Neural correlates of multi-day learning and savings in sensorimotor adaptation

M.F.L. Ruitenberg [1,a], V. Koppelmans [1,b], Y.E. De Dios [2], N.E. Gadd [2], S.J. Wood [3], P.A. Reuter-Lorenz [4], I. Kofman [2], J.J. Bloomberg [3], A.P. Mulavara [2], & R.D Seidler [1,4,c] *

[1] School of Kinesiology, University of Michigan, Ann Arbor, MI, USA.

[2] KBRwyle Science, Technology, and Engineering Group, Houston, TX, USA.

[3] NASA Johnson Space Center, Houston, TX, USA.

[4] Department of Psychology, University of Michigan, Ann Arbor, MI, USA.

Current affiliations:

[a] Department of Experimental Psychology, Ghent University, Ghent, Belgium

[b] Department of Psychiatry, University of Utah, Salt Lake City, UT, USA

[c] Department of Applied Physiology & Kinesiology, University of Florida, Gainesville, FL, USA

* Corresponding author:

Rachael Seidler

University of Florida, Department of Applied Physiology & Kinesiology

1864 Stadium Road, Gainesville, FL 32611-8205, USA

Telephone: +1 (352) 294-1722

Email: rachaelseidler@ufl.edu

Supplementary Figure S1. Exemplary single subject data and fitted exponential decay functions for the early and late adaptation phases.

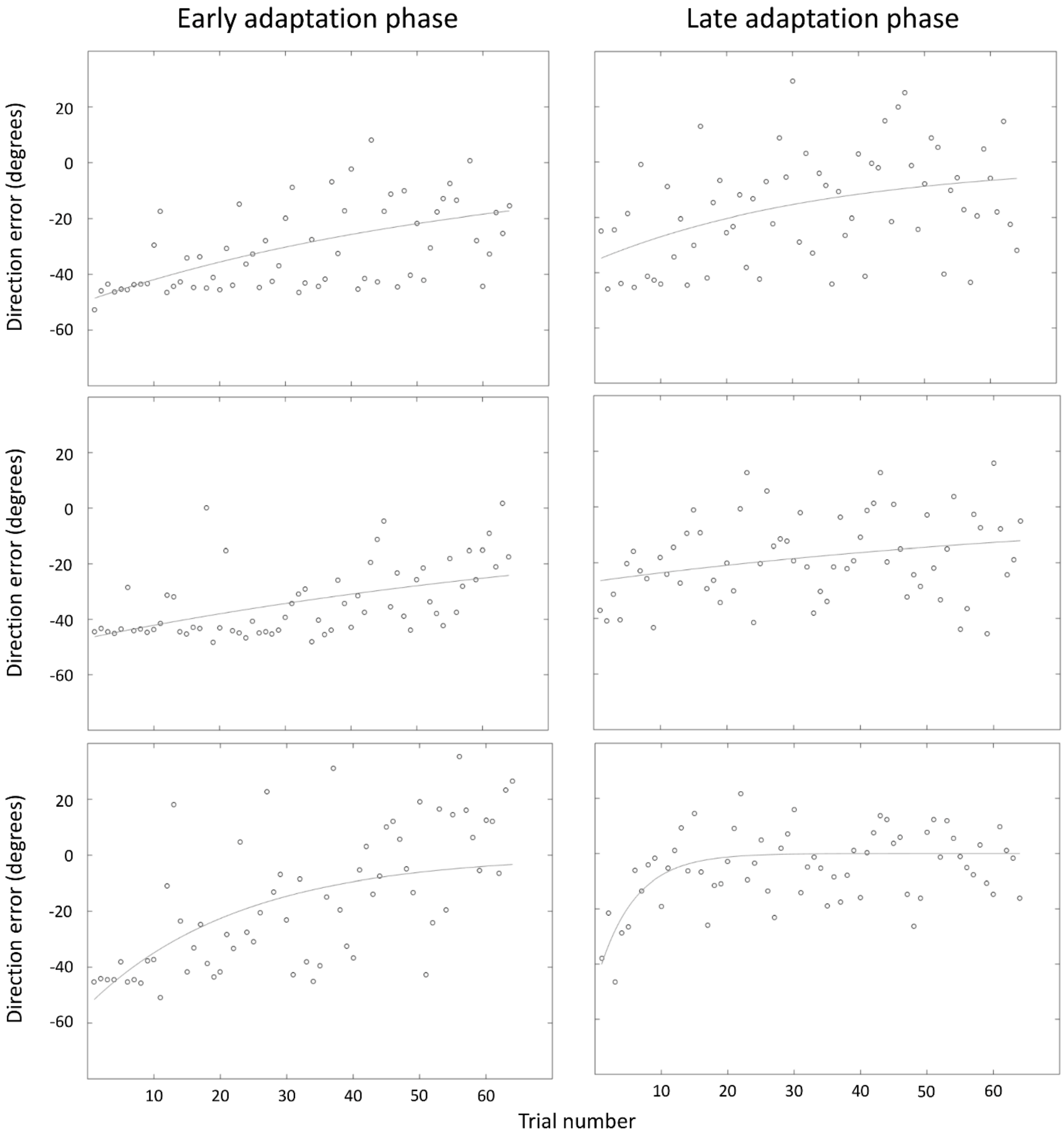

Supplement: Supplementary file 1 — Supplementary Material [file 41598_2018_32689_MOESM1_ESM.pdf]
